# Framing utility: Regulatory reform and genetic tests in the USA, 1989–2000

**DOI:** 10.1016/j.socscimed.2020.112924

**Published:** 2022-07

**Authors:** Steve Sturdy

**Affiliations:** Science, Technology and Innovation Studies, University of Edinburgh, Old Surgeons' Hall, High School Yards, Edinburgh EH1 1LZ, UK

**Keywords:** United States, Genetic tests, Regulation, Clinical utility, Genetic exceptionalism, Genetic counselling, Eugenics

## Abstract

Before about 1990, insofar as diagnostic and other medical tests were subject to regulatory oversight, it was chiefly to ensure that they met appropriate standards of analytic and clinical validity. Over the course of the 1990s, however, regulatory reformers in the United States began to argue that genetic tests, specifically, should also be assessed to determine whether or not they actually benefit those undergoing testing—whether they possess “clinical utility”, as they put it. The present paper asks why this shift in regulatory focus occurred specifically in relation to genetic tests, and why clinical utility became a key object of assessment. It answers these questions by situating concerns about genetic tests in the longer history of medical genetics. Looking back to the 1970s and medical geneticists' efforts to distance themselves from their earlier association with eugenics, it shows that they adopted a particular framing of the dangers of genetic testing which would inform their response to the proliferation of new genetic tests and the growth of commercial testing in the 1990s. In a series of policy committees convened over the course of that decade, medical geneticists called for regulatory measures to be implemented to ensure that genetic tests were only introduced into medical practice if they had been shown to be beneficial to those tested. The paper follows the deliberations of those committees to show in detail how geneticists worked within this framing to accommodate new technical capacities and regulatory opportunities. In the course of these deliberations, they adopted the idea of clinical utility to signify the need for evidence of benefit specifically to those tested. The paper concludes with some observations regarding how this framing of genetic tests relates to current understandings of “genetic exceptionalism” and to more recent articulations of clinical utility.

## Highlights

• Calls to regulate genetic tests on grounds of “clinical utility” first emerged in the 1990s.
• They were informed by a framing of the risks of genetic testing that dated back to the 1970s.
• That framing stressed the need for tests to benefit the individuals undergoing testing.
• Calls for formal regulation reflected geneticists' declining control over genetic testing.
• More recent versions of “clinical utility” neglect the need for testing to benefit those tested.

## 1 Introduction

Before about 1990, insofar as diagnostic and other medical tests were subject to statutory regulation, it was chiefly to ensure that they met appropriate standards of accuracy—of analytic and clinical validity, in present-day terminology. Other than that, regulatory agencies generally assumed that medical practitioners were best left to decide for themselves, on the basis of their own knowledge and experience, whether and when to use such tests. Over the course of the 1990s, however, American medical geneticists began to argue that genetic tests, specifically, should also be assessed to determine whether or not they actually benefit those undergoing testing—whether they possess what came, in the course of these debates, to be called “clinical utility”. While these efforts had only limited impact on the statutory regulation of genetic tests, the idea of assessing their clinical utility has been widely adopted in the formulation of professional clinical guidelines, as well as by health technology assessment bodies charged with deciding whether new genetic tests are worth implementing in practice. The turn to utility as a key assessment criterion thus represents a significant shift in ideas about the extent of medical competence and the role of regulation in relation to genetic testing.

How and why concerns first came to be expressed about the regulation of genetic tests, and especially about their utility, remains under-explained, however. The only author to address these questions at any length is STS scholar Shobita Parthasarathy, in the first chapter of her book *Building Genetic Medicine* (Parthasarathy, 2007, pp. 28–45). Parthasarathy attributes these concerns to the development, from the early 1990s, of new genetic tests made possible by novel genomic biotechnologies. Medical geneticists feared that such tests would be used by practitioners who lacked specialist expertise in interpreting and communicating genetic risks to their patients, and that this would do more harm than good to patients, particularly when effective treatments were lacking. It was this, argues Parthasarathy, that led medical geneticists to demand more stringent controls over the marketing and use of such tests.

Parthasarathy's analysis leaves two questions unanswered, however. First, why did such concerns focus specifically on genetic tests, and not on other kinds of diagnostic and predictive tests that were becoming available at the same time? Other novel medical biotechnologies—notably monoclonal antibodies—were at least as fertile a source of new diagnostic and predictive tests, many of which involved similar problems of risk communication to predictive genetic tests. Yet while some practitioners expressed doubts about the possible harms of testing more generally, they did not voice the same kinds of calls for regulatory oversight as accompanied genetic testing. Why, then, were calls for enhanced regulatory oversight of clinical tests associated specifically with genetic tests? Secondly, why did calls for regulation of genetic tests come to focus on assessment of their clinical utility, and what, in that setting, did “clinical utility” actually mean?

In the present paper I seek to answer this question by situating medical geneticists' concerns about genetic tests in the longer history of their specialism. As Parthasarathy observes, “the architecture of a genetic test was not simply a collection of laboratory practices and machines, but also included clinical care such as how information was transmitted to clients and what types of medical interventions had been devised to deal with at-risk status” (Parthasarathy, 2007, p. 42). The present paper historicises this observation. Informed by frame theory (Rein and Schön, 1996; Jones, 2001), the paper shows that medical geneticists' sensitivity to the informational dynamics of genetic testing dated back at least to the 1970s, when they sought to distance themselves from their earlier association with eugenics; that the way they framed the risks associated with genetic tests at that time continued to inform their responses to the development of new genetic diagnostics during the 1990s; and that the idea of “clinical utility” as a key regulatory requirement for genetic tests emerged as geneticists sought to maintain and adapt that problem frame to accommodate new developments both in diagnostic technology and in the regulatory environment. It concludes with some observations regarding how this framing of genetic tests relates to current understandings of “genetic exceptionalism” and to more recent articulations of clinical utility in the regulation and governance of testing.

The paper is based on a contextual reading of medical geneticists' views about genetic testing, the possible harms it posed, and how to avoid those harms, from 1989 to 2000. The data are mostly though not exclusively drawn from published sources. For the period 1989–1993, when geneticists were first taking stock of the implications of new DNA-based testing technologies, the paper draws on geneticists' arguments and opinions published in medical and scientific periodicals, as well as looking back to the 1975 report of a committee convened by medical geneticists where their prevailing framing of genetic testing was previously articulated. For the period 1993–2000 it draws chiefly on the published reports and recommendations of a series of advisory committees in which leading medical geneticists were able to air their considered opinions about genetic tests and their proposals for regulation. It also uses materials in the Archives of the National Academy of Sciences to throw light on the background and aims of the Institute of Medicine Committee on Assessing Genetic Risks. The views expressed in these sources were analysed to identify, on the one hand, continuities in geneticists' overall framing of the problems associated with testing, and on the other hand, the adjustments made over time to accommodate new technical and regulatory developments within that overall framing.

### 1.1 Cystic fibrosis carrier screening

In September 1989, the leading journal *Science* published a series of papers that quickly became a milestone in disease genetics. Researchers had located and partially sequenced a gene on human chromosome 7 which they called “the cystic fibrosis gene”. They also identified a mutation—ΔF508—in that gene that accounted for a large proportion of cystic fibrosis cases (Rommens et al., 1989; Riordan et al., 1989; Kerem et al., 1989). Their work created dramatic new possibilities for medical efforts to tackle cystic fibrosis.

Cystic fibrosis (CF) has been understood from the 1950s to be a recessive single-gene disorder. Individuals who inherit two defective copies of the gene—one from each parent—develop the disease; while those who inherit only a single defective copy are unaffected carriers. This knowledge offered limited opportunities for medical intervention. In families where medical geneticists were able to map the inheritance of the disease, they could often calculate the probability that any given individual within that family was a carrier, and so could advise that individual on the risks of having an affected child. Given the rarity of CF mutations in the general population, however, most cases were diagnosed before any family history of the disease became evident. Consequently, efforts to mitigate the effects of CF generally focused on improving treatment options for children already diagnosed with the condition, rather than on its genetic aspects (Wailoo and Pemberton, 2008, pp. 68–91).

Medical interest in the genetics of CF gained ground during the 1980s, spurred by developments in molecular genetics. By the middle of the decade, researchers had mapped a number of DNA markers showing linkage to the putative CF gene. Observing the transmission of these markers from one generation to the next made it possible to determine with much greater certainty which individuals in an affected family were carriers—but still only in “informative” families, i.e. those with a sufficiently well-document history of cystic fibrosis and with a suitable marker (Ostrer and Hejtmancik, 1988; Johnson, 1988). The cloning of “the cystic fibrosis gene” itself, and the characterisation of the ΔF508 mutation, greatly simplified the testing process. Moreover, it made it possible to test anyone for ΔF508 carrier status, irrespective of their family history. Some medical geneticists immediately hailed this as an opportunity to undertake population-wide screening to identify CF carriers (e.g. Schulman et al., 1990; cf. Kerr, 2005, p. 882). But others urged caution (e.g. Kerem et al., 1989, p. 1079; Gilbert, 1990; Wilfond and Fost, 1990). Their ambivalence was informed by almost twenty years of debate about the risks and benefits of genetic screening, particularly in the United States.

In 1972, responding to calls from leading figures in the American Society of Human Genetics (ASHG), the US National Academy of Sciences established a Committee for the Study of Inborn Errors of Metabolism—a large class of usually single gene disorders, many of which become apparent quite early in life. Prompted by questions about the effectiveness of newborn screening for phenylketonuria, which had been rolled out—often on a mandatory basis—in a growing number of American states since the mid-1960s, as well as by controversies about screening for sickle-cell anaemia, Tay-Sachs disease, and birth defects such as Down syndrome, the Committee was charged to report on “the problems and difficulties [arising from these initiatives] and give some procedural guidance, in order to minimise the shortcomings and maximize the effectiveness of future genetic screening programs” (CSIEM, 1975, p. iii; Paul and Brosco, 2013, pp. 63–91, 96–97; Lindee, 2005, pp. 28–57).

The Committee's response was coloured by geneticists' desire to distance themselves from controversial questions of social policy, especially their earlier association with eugenics (Mitchell, 2017). Consequently, their recommendations reflected a strong commitment to safeguarding the rights and interests of those who underwent screening. In particular, where earlier eugenic programmes had cast genetics as a tool for paternalistic and coercive control of human reproduction, the Committee now argued that individuals should be encouraged and enabled to use genetics to inform their own reproductive decisions. Consequently, “Participation in a genetic screening program should not be made mandatory by law, but should be left to the discretion of the person tested”. Screening agencies should take appropriate steps “to avoid social consequences of screening that may be damaging”, such as “invasion of privacy, breach of confidentiality … as well as psychological damage resulting from being ‘labeled’ or from misunderstandings about the significance of diseases and carrier states”. To that end, it was crucial to ensure that “qualified and effective counselors are available in sufficient number” (CSIEM, 1975, pp. 1–4).

The Committee's recommendations reflect a particular framing of the predicament in which clinical geneticists found themselves in the early 1970s. While new tools were becoming available to identify and intervene in certain genetic disorders, geneticists were acutely aware that their long association with eugenics could provoke suspicion about their motives. The Committee framed the problem as in effect one of trust, and the solution in terms of a redistribution of control. As far as possible, control over the process of testing, and over any decisions arising from it, should rest, not with geneticists or the state, but with the individuals being tested. Hence geneticists' opposition to mandatory testing. Hence too their emphasis on counselling, and specifically on non-directive counselling. Estimations of genetic risk were highly technical in nature, based in abstract theories of inheritance and epidemiology and usually expressed as probabilities rather than certainties. As such, they could easily leave those tested feeling confused, anxious, and disempowered. Genetic counselling developed as a set of interpretative and communicative practices intended to help individuals understand such information in a way that made sense in the context of their own lives, and to support them as they made their own life choices (Stern, 2012; Paul, 1997).

The same framing informed medical geneticists' response to the possibility of carrier screening for cystic fibrosis. It focused attention on the informational uncertainties inherent in the available tests, and the harms they might cause to those tested and to medical genetics itself. The ΔF508 mutation only accounted for around 70% of all CF carriers, with the remaining 30% carrying as yet unidentified mutations. A test which detected 70% of CF carriers would identify only 50% of couples in which both partners carried a deleterious mutation, and who were therefore at risk of having an affected child. A significant number of affected children would consequently be born to parents one or both of whom had received negative carrier test results. Moreover, in about one in every fifteen couples across the entire US population, one partner would test positive and the other negative. Since the partner who tested negative might still carry a deleterious mutation, these couples would learn that, relative to the rest of the population, they were “at increased risk (approximately 1 in 500) of bearing a child with cystic fibrosis” (National Institutes of Health, 1990, p. 70). But what should they make of that information? Far from clarifying their reproductive options, there was a danger that carrier screening would leave them in “genetic limbo”, in the evocative phrase of one science journalist (Roberts, 1990, p. 1297).

Genetic counselling would help those who underwent cystic fibrosis carrier testing to make sense of their results. But the sheer volume of testing that would result from population-wide CF carrier screening would far exceed the capacity of existing genetic services to provide such counselling. As early as 1983, when the development of cystic fibrosis carrier tests first began to look like a realistic prospect, medical geneticists had warned that “If a test becomes available to identify these carriers, the demand for genetic screening and counselling could quickly become overwhelming” (President's Commission, 1983, p. 5). With the identification of the cystic fibrosis gene and the development of a test for ΔF508, these warnings acquired a new urgency. If screening for ΔF508 were rolled out population-wide, one analysis observed, “the usual standard of care in genetic counselling will not be feasible” (Wilfond and Fost, 1990, p. 2781. Also e.g. Biesecker et al., 1992). And in the absence of proper counselling, medical geneticists feared that those tested would suffer anxiety, distress, and the risk of reproductive outcomes they might have preferred to avoid. This had implications, not just for those who underwent screening, but for the very enterprise of medical genetics. As one geneticist put it, carrier screening could prove “a mistake and a disservice to the clinical genetics community” due to the “significant false negative rate. The potential problems that this may cause, in counselling and medical liability, are enormous. The possibility that the public's perception, or acceptance, of genetic testing may be negatively affected by unrealistic expectations of such testing is real” (Gilbert, 1990, p. 394).

Given these concerns, in November 1989 the American Society of Human Genetics (ASHG) adopted a statement that while “it will be appropriate to begin large-scale population screening in the foreseeable future”, it should not be rolled out until “the test detects a larger proportion of CF carriers and more information is available regarding the issues surrounding the screening process” (Caskey et al., 1990). By the spring of 1992, enough additional mutations had been identified to raise the carrier detection rate to almost 90%. But even this could only mitigate the problem, not eliminate it. The ASHG therefore repeated its warning that “CF testing is not recommended, at this time, for individuals or couples who do not have a family history of CF”, and advised that population screening be further deferred until a series of pilot studies had been completed (American Society of Human Genetics, 1992). The Society's position was endorsed by the American Medical Association and the American College of Obstetricians and Gynecologists among others, as well as by special workshops convened by the National Institutes of Health (NIH) and by the NIH/Department of Energy Joint Working Group on the Ethical, Legal and Social Implications of Human Genome Research (Wilfond and Nolan, 1993, pp. 2951–2952; Cook-Deegan, 1994, pp. 241–246). In view of this concerted opposition, population screening for cystic fibrosis carriers remained on hold, at least for the time being.

### 1.2 The turn to regulation: CLIA88

While professional self-restraint was sufficient to stall the introduction of cystic fibrosis carrier screening, medical geneticists also began exploring other, more formal means of regulating genetic tests. Besides calling for deferral of population screening, the ASHG's 1989 statement on CF carrier testing also declared “an immediate need for centralized quality control of laboratories conducting these tests” (Caskey et al., 1990). In so doing, it invoked another debate under way at that time about the regulation, not specifically of genetic tests, but of medical tests more generally.

In the mid-1980s, the *Wall Street Journal* and other US media outlets ran a series of reports about laboratories returning unacceptably high rates of inaccurate or misleading results for cervical smears and other medical tests. In 1988, Congress responded by passing the Clinical Laboratory Improvement Amendments (CLIA88), which aimed to strengthen and extend powers vested in the US Public Health Service, acting through the Centers for Disease Control (CDC), to impose quality assurance procedures on laboratories offering medical testing services (Peddecord and Hammond, 1990). The legislation prompted an extended process of consultation by the Health Care Financing Administration (HCFA)—the Federal agency responsible for implementing CLIA88—over just how the legislation should be implemented.

Alerted by their concerns about cystic fibrosis testing, clinical geneticists were among those who responded to the consultation. Their response was informed by their distinctive framing of the potential harms posed by genetic tests, and of the kinds of measures needed to mitigate those harms. As originally conceived, CLIA88 focused primarily on ensuring that test results met appropriate standards of accuracy and reliability. The statute therefore identified proficiency testing—direct monitoring of a laboratory's performance—as “the central element in determining a laboratory's competence” (quoted in Peddecord and Hammond, p. 2032), along with more stringent staffing requirements to ensure that laboratories possessed the technical expertise to conduct tests accurately. As we have seen, however, clinical geneticists took the view that accurate test results could still be harmful if incorrectly interpreted or understood. Their submissions to the consultation reflected this. As Thomas Caskey and Herbert Lubs of the ASHG observed, genetic tests have “unique features which require interpretation of data on a highly individualized basis for a specific family or patient. Detailed information about a specific patient or family, and a complex, computerized program is often required for such interpretation”. Consequently, they urged, laboratories undertaking genetic tests should be required to employ staff with recognised competence in medical genetics, so that test results could be accompanied by a proper interpretation of those results; while proficiency testing of participating laboratories should examine not just the analytical accuracy of test results but also the quality of the interpretations that accompanied them (House Subcommittee on Human Resources and Intergovernmental Relations, 1993, pp. 107–108; see also House Subcommittee on Human Resources and Intergovernmental Relations, 1993, pp. 115–122).

Clinical geneticists got an opportunity to put these view directly to legislators in July 1992, when the House of Representatives Subcommittee on Human Resources and Intergovernmental Relations held a special hearing to consider issues in human genetics that remained unresolved under CLIA88. Introducing the hearing, the chair, Representative Ted Weiss, noted that genetic test results sometimes “cause needless fear and frustration”, and proposed that measures were needed “to protect patients, and society as a whole, from the risks inherent in genetic testing” (House Subcommittee on Human Resources and Intergovernmental Relations, 1993, p. 2). Three expert witnesses from the world of clinical genetics—paediatrician-geneticist Tony Holtzman, genetic counsellor Elizabeth Gettig and geneticist Paul Billings—recounted instances of patients who had suffered anxiety, and sometimes unnecessary medical interventions, as a result of genetic tests. Increasingly, they argued, family physicians were commissioning a growing range of genetic tests directly from commercial laboratories. Often, neither the physician nor the laboratory possessed the expertise to counsel those tested on the meaning of the results and the courses of action open to them—hence the anxiety they suffered. To avoid such problems, Gettig argued, “medical geneticists should be directly involved with reporting of genetic laboratory tests. To have a person with this specialized training interpret genetic testing is essential” (House Subcommittee on Human Resources and Intergovernmental Relations, 1993, p. 24). To that end, Holtzman proposed that genetic tests be recognised under CLIA88 “as a separate category requiring its own standards to assure high quality of laboratory use”, and reiterated ASHG's recommendation that these standards “must apply to laboratories' interpretations of test results … as well as … their performance of the test” (House Subcommittee on Human Resources and Intergovernmental Relations, 1993, p. 83).

### 1.3 Regulatory reform and the institute of Medicine Committee

This same framing of the need for special regulatory measures for genetic tests would also inform the deliberations of a series of policy committees over the course of the 1990s. The first of these was convened by the Institute of Medicine (IOM)—part of the National Academy of Sciences—which in May 1990 set about establishing “a panel of experts to evaluate issues in the development, application, and use of tests for genetic disorders”. In proposing this enquiry, the IOM was moved by many of the same concerns that medical geneticists had raised about cystic fibrosis carrier screening. Where medical genetics had previously dealt primarily with individuals “known to be at risk because of their family history”, the IOM anticipated that developments in molecular genetics would soon make it possible “to detect abnormal genes directly, opening the floodgates for predictive genetic screening for the masses”. Among the diseases the IOM anticipated would be brought within the scope of predictive genetic testing were not just cystic fibrosis, but “schizophrenia, alcoholism, and certain cancers”—common health problems, with a far higher incidence than the rare monogenic conditions and birth defects that had previously occupied medical geneticists. “If these tests become more widespread,” the IOM noted, “it is doubtful that there will be enough health care professionals prepared to provide even the most basic counselling required to assist patient understanding” (National Academy of Sciences, Institute of Medicine, 1990).

The IOM did not initially include laboratory regulation among the topics to be considered by the Committee. But when the prospective members—mostly clinical and molecular geneticists, plus a number of lawyers, ethicists, and members of patient and civil society organisations—were canvassed on their views, it became apparent that they regarded “laboratory quality assurance, including the clouded regulatory climate” as a “focal issue” (Fullarton, 1991). The Committee duly added it to their agenda, holding “meetings with federal officials responsible for implementing federal regulations under … CLIA88” (IOM, Committee on Assessing Genetic Risks, 1994, pp. 10–11). Its report, published in 1994, echoed the recommendations that had been put to the House Subcommittee two years earlier. Noting that “existing CLIA88 regulations … are not being applied to genetic testing at all”, the Committee again urged that the rules be revised to incorporate special requirements for genetic testing laboratories, including employment of appropriately qualified laboratory personnel, and regulatory oversight of the interpretation as well as the accuracy of test results (IOM, Committee on Assessing Genetic Risks, 1994, pp. 11; 136–138).

CLIA88 was a late addition to the Committee's agenda. But the IOM had another set of regulatory provisions in its sights from the start. Among the topics initially proposed for consideration was “the ability of the Federal [sic.] Drug Administration (FDA) to guide the evaluation of the efficacy, sensitivity and reliability of tests given the unique complexity of genetic disease” (National Academy of Sciences, Institute of Medicine, 1990). This opened a second line of approach for the geneticists on the Committee to engage with regulatory questions, while maintaining the same overall framing of the issues.

The US Food and Drug Administration had long been empowered to regulate the marketing of diagnostic tests, but exercised those powers with a notably light touch. FDA confined its regulatory oversight to tests sold as kits and to commercially-marketed reagents, leaving tests sold as laboratory services to be regulated under CLIA. And even where test kits were concerned, FDA sought as far as possible to work cooperatively with manufacturers rather than impose strict standards (Merrill, 1996, p. 1812). In practice, diagnostic tests often reached the market after only the most cursory regulatory scrutiny. In the case of “genetic testing kits and associated genetic test reagents and DNA probes,” the IOM Committee noted in its 1994 report, “such tests are rarely being submitted to FDA for approval.” Given the risks they associated with genetic tests, the Committee considered this unsatisfactory. Consequently, they recommended that FDA use the powers at its disposal to ensure the “safety and effectiveness” of genetic tests by requiring all new genetic tests to undergo full premarket assessment procedures (IOM, Committee on Assessing Genetic Risks, 1994, pp. 11–13). More than this, however, in keeping with their framing of the distinctive informational risks associated with genetic tests, they recommended that FDA also require manufacturers to provide significantly more information about their tests than was demanded for other kinds of diagnostic devices.

Manufacturers seeking approval for a diagnostic test were normally expected to provide evidence that it accurately measured what it claimed to measure, that the results were “clinically significant”, and that the numbers of false positive and false negative results it generated were within acceptable limits. They were not usually expected to provide information about how a test should be used (Gutman, 1999, pp. 747–748). This was a matter of deliberate FDA policy. Congress had repeatedly declared that the legislation authorising FDA to regulate medical products was not intended to regulate medical practice. FDA interpreted this to mean that they should not seek to influence how doctors chose to use diagnostic devices (Huang, 1998, p. 574; Evans, 2006, p. 775). In 1987, for instance, discussing FDA's role in relation to new DNA tests for infectious disease agents, FDA Commissioner Frank Young had stated: “The FDA … cannot decide for practitioners when a test is appropriate, and under what circumstances any particular test should be used … The FDA does not have or should not have a direct regulatory role in the practice of a physician.” Rather, it was up to medical professionals to “learn more about the utility and reliability of tests, know and contact medical specialty colleagues familiar with the tests; be familiar with the technical literature; and attend professional meetings” (Young, 1987, p. 2405). So far as FDA were concerned, medical professionals could be expected to possess or acquire the expertise they needed to use it safely and effectively.

This was precisely what medical geneticists doubted: most practitioners, they feared, were simply not equipped to interpret and communicate the results of genetic tests in ways which did not endanger their patients. The IOM Committee therefore recommended that FDA's premarket approval procedures for genetic tests should be enhanced to minimise that risk. Manufacturers should be required to indicate “the intended and potential use(s) of the test (e.g., presymptomatic diagnosis or prediction, carrier screening, prenatal diagnosis)”, and to provide relevant data “for each intended use”. Additionally, manufacturers should provide a “description to be given to health care providers and to patients regarding the objectives of the test and the interpretations of negative or positive findings” (IOM, Committee on Assessing Genetic Risks, 1994, pp. 139–140). In effect, FDA's premarket approval procedures should be revised to ensure practitioners received guidance on when and how to use genetic tests and how to interpret the results to their patients.

The IOM Committee thus developed a two-prong regulatory strategy to address the problems it associated with genetic tests, using both CLIA88 and FDA's medical device regulations. The members would have been well aware that the regulatory agencies were unwilling to take on additional responsibilities, however. At the same July 1992 meeting of the House Subcommittee on Human Resources and Intergovernmental Relations addressed by Holtzman, Gettig and Billings, representatives from CDC, HCFA and FDA also attended to answer questions about their oversight of genetic tests. Their testimony was not encouraging. Asked about plans to implement CLIA88, the HCFA representative revealed that no measures were being taken to ensure that laboratories employed personnel with the expertise to interpret genetic tests, while proficiency testing was awaiting the formulation of programmes that HCFA deemed could be rolled out nationally; asked whether, in the specific case of cytogenetics, this was likely to take years or decades, the CDC representative declined to offer an estimate (House Subcommittee on Human Resources and Intergovernmental Relations, 1993, pp. 123–129). As for FDA regulation of genetic tests: the FDA representative said that the agency was aware that many test kits were being used for purposes that had not been approved, and declared that FDA had recently “put the industry on notice” that such use needed to be brought under control. However, the representative noted, the tests had already “reached a level of clinical acceptability”, and FDA was anxious to “avoid completely upsetting the current status of the use of the testing” so that “reasonable medical use of these products is not destroyed” (House Subcommittee on Human Resources and Intergovernmental Relations, 1993, pp. 130–132). The chair could only express his exasperation. “The FDA was created not for the benefit of manufacturers; the FDA was created for the benefit of the American people”, he complained. “When the [regulatory] process gets in the way of protecting their health and safety and allows for patients to be given misinformation, then the agency charged with protecting the public is misusing its mandate from Congress.” (House Subcommittee on Human Resources and Intergovernmental Relations, p. 133).

### 1.4 Towards utility: the NIH-DOE Task Force and SACGT

Despite heel-dragging by the regulatory agencies, the IOM Committee's framing of the need for stricter regulation of genetic testing struck a chord, not least among proponents of the human genome project. Much of the funding for the IOM Committee had come from the joint National Institutes of Health (NIH) and Department of Energy (DOE) budget for research into the ethical, legal and social implications (ELSI) of human genome science. Following publication of the IOM Committee's report, the NIH-DOE ELSI Working Group decided to set up its own Task Force on Genetic Testing to conduct further investigations and “when necessary, make recommendations to ensure the development of safe and effective genetic tests” (Task Force on Genetic Testing, 1997, ch. 1). The Task Force published its draft recommendations for comment in January 1997 (National Institutes of Health, 1997), and released its Final Report in the following October. Among its recommendations, the Task Force proposed that the Secretary of State for Health and Human Services appoint her own expert committee to advise on policy development. The Secretary's Advisory Committee on Genetic Testing (SACGT) was duly chartered in June 1998 (National Institutes of Health, Office of Science Policy, n.d.), and released its first set of proposals for “Enhancing the oversight of genetic tests” in July 2000 (SACGT, 2000).

The successive committees charted a trajectory through the orbits of government policy-making, from the academic detachment of the IOM committee to SACGT in the office of the Secretary of Health and Human Services. At the same time, the balance of interests represented in the committees shifted to include a larger contingent of stakeholders and policy experts (Parthasarathy, 2007, p. 40). By the time the SACGT was appointed, medical geneticists and genetic counsellors were in a minority. Nonetheless, the chair continued to be held by a medical geneticist, and the overall framing of the issues around genetic tests remained largely constant. Like the IOM committee, the Task Force and SACGT maintained that genetic tests needed to be properly interpreted and communicated if they were not to harm those undergoing testing, and feared that the proliferation of such tests beyond the immediate interpretative control of medical geneticists posed a real risk. However, the precise locus of concern within that frame shifted somewhat from the IOM Committee to the Task Force then the SACGT, as did the relative weight the committees gave to different kinds of regulatory solutions.

On the one hand, the Task Force and the SACGT were more sanguine than the IOM committee about the ability of non-geneticists to make safe use of genetic tests. “Even when aware that a problem that concerns them might have a genetic origin, they [patients] are more likely to seek the care of the specialist who manages the problem when it becomes overt than the care of a geneticist”, noted the Task Force. “Consequently, non-genetic specialists, as well as primary care providers, become the gateway to genetic testing.” But far from being feared, this should be welcomed. “With proper training and adequate knowledge of test validity, disease and mutation frequencies in the ethnic groups to whom they provide care, primary care providers and other non-genetic specialists can and should be the ones to offer predictive genetic tests to at-risk individuals”. Measures would need to be put in place to ensure that practitioners received the necessary training, including not just technical knowledge of genetics and genetic tests, but also a practical appreciation of “the means of communicating genetic concepts and risks to patients”. But the Task Force noted that such measures were already being implemented by a range of professional and policy bodies, and simply urged that these measures be encouraged and supported (Task Force on Genetic Testing, 1997, ch. 4).

On the other hand, the committee reports reflected growing concern at the accelerating commercialisation of genetic tests and testing. “Until the 1980s”, the Task Force observed, “most genetic and cytogenetic testing was performed in the laboratories of non-profit organisations, most of them in academic medical centers. These labs were often directed by the same professionals who cared for patients. In the last decade, genetic testing has been commercialized. As a result, providers who were close to patients and families at risk of illness might not have as much influence on testing policy as they once did” (Task Force on Genetic Testing, 1997, ch. 1). Geneticists worried that, in the absence of professional oversight, companies might market their tests without regard for the possible harms to patients—a fear that was boosted from the mid-1990s as biotechnology company Myriad launched an aggressive programme of direct-to-consumer advertising and market monopolisation of its tests for the breast cancer susceptibility genes BRCA1 and BRCA2 (Malinowski and Blatt, 1997; Parthasarathy, 2007, pp. 58–92; Baldwin and Cook-Deegan, 2013).

As the committees' anxieties shifted to focus on the expanding commercial sector, so they looked increasingly to external regulatory agencies to mitigate those anxieties. This included repeating the IOM committee's calls to strengthen oversight of genetic testing laboratories under CLIA88: laboratories should be required, among other things, to return genetic test results “in a form that is understandable to the non-geneticist health care provider”, and to employ staff with adequate training and experience in genetics to provide such interpretations (Task Force on Genetic Testing, 1997, ch. 3). The relevant agencies appeared increasingly willing to implement such changes. By the time SACGT reported in 2000, HCFA and CDC were “taking steps to develop more specific laboratory requirements for genetic testing under CLIA, including provisions for the pre- and post-analytical phases of the testing process” (SACGT, 2000, p. 9). This included drafting proposals that laboratories should not just report the results of genetic tests, but should also provide an interpretation; and that laboratories be required to employ staff “capable of providing genetic counselling to the laboratory's clients (care providers, patients, individuals, etc.)” (Centers for Disease Control and Prevention, 2000, p. 25932).

The Task Force and SACGT also called for FDA to do more to regulate genetic tests. On this issue, they went significantly beyond what the IOM committee had recommended. As we have seen, the IOM Committee had proposed that FDA's premarket approval procedures be expanded to include information about the intended use of genetic tests and about how to interpret the results. However, there are indications that the IOM Committee was leaning towards stronger regulatory measures. FDA approval of diagnostics and other devices was generally understood to depend on evidence of “safety and effectiveness”. Twice in its report, however, the IOM Committee referred instead to the need to assess the “safety, effectiveness, *and clinical utility*” of genetic tests, and particularly screening tests (IOM, Committee on Assessing Genetic Risks, 1994, pp. 13; 142, emphasis added). It also observed that tests for inherited predispositions to common disorders such as heart disease and cancer would raise additional regulatory challenges, since such disorders “will vary in treatability, thereby affecting the utility of the information to be gained even from highly predictive tests” (IOM, Committee on Assessing Genetic Risks, 1994, p. 296). The Committee nowhere specified what it meant by “clinical utility”. However, the fact that it arose specifically in relation to screening tests and the availability of effective treatment recalls geneticists' long-standing framing of the harms that ill-considered or badly administered genetic tests could cause. It also indicates that geneticists were beginning to consider additional ways of mitigating such harms: where previously they had focused primarily on the communicative technology of genetic counselling, they were now thinking also of how regulatory agencies, and especially FDA, could act to ensure that tests were delivered in ways that brought benefit rather than harm to those tested.

This became explicit in the deliberations of the Task Force. “Before a genetic test can be generally accepted in clinical practice, data must be collected to demonstrate the benefits and risks that accrue from both positive and negative results,” it declared. To that end, tests should be assessed according to three key criteria: analytic validity, clinical validity, and clinical utility. Premarket approval procedures should therefore include data on “the test's *utility for individuals who are tested*”; while “the clinical use of a genetic test must be based on evidence that … the test results will be *useful to the people being tested*” (Task Force on Genetic Testing, 1997, ch. 2, emphasis added). SACGT concurred, while adding a fourth criterion of evaluation: “Analytical validity, clinical validity, clinical utility, and social consequences should be the major criteria used to assess the benefits and risks of genetic tests”, declared the committee (SACGT, 2000, p. viii). FDA should therefore “delineate review processes for pre-market evaluation of genetic tests. These processes should focus on evaluation of the data regarding analytical and clinical validity, as well as on claims made by the developer of the test about its clinical utility” (SACGT, 2000, p. x).

Both committees also inclined to the view that this should apply, not just to the test kits and reagents that FDA currently chose to regulate, but also to tests sold as laboratory services. As the Task Force noted, “Under the CLIA, clinical laboratories must demonstrate analytical validity of their tests but there is no statutory or regulatory requirement for them to establish the clinical validity or utility of clinical laboratory tests” (National Institutes of Health, 1997, p. 4544). Consequently, the Task Force initially proposed that FDA expand its remit by requiring “*all* organisations developing new, predictive genetic tests” to undergo full premarket assessment, “regardless of whether their sponsor's intention is to market [those tests] as services or as kits” (National Institutes of Health, 1997, pp. 4541, 4544). However, a minority of Task Force members dissented from this proposal (National Institutes of Health, 1997, p. 4544), which was replaced in the final report with a recommendation that “Testing organisations should comply voluntarily” with this requirement (Task Force on Genetic Testing, 1997, ch. 2). SACGT showed no such ambivalence, stating categorically that “FDA should be the federal agency responsible for the review, approval, and labeling of all new genetic tests that have moved beyond the basic research phase” (SACGT, 2000, p. x), including tests marketed as services as well as those sold as test kits (SACGT, 2000, p. 10).

## 2 Discussion

The idea that genetic tests should be assessed for utility before being approved for clinical practice took shape over the course of the 1990s in a series of policy committees beginning with the IOM Committee on Assessing Genetic Risks and ending with the Secretary's Advisory Committee on Genetic Testing. It was informed by medical geneticists' distinctive framing of genetic tests and the harms they could pose, both to those tested and to the enterprise of medical genetics itself. That framing originated in the 1970s, driven by geneticists' concern to counter public mistrust due to their long association with eugenics, and continued to shape their attitudes to testing through the 1990s. Within that framing, however, geneticists adjusted their ideas about how best to mitigate the potential harms of genetic testing in response to changing circumstances. Initially they concentrated on keeping genetic testing procedures under their own specialist control, subject to skilled genetic counselling. But by the mid-1990s, they were coming to accept that more and more genetic tests would be marketed by commercial organisations and delivered by practitioners lacking any special expertise in genetics. Faced with their inability to control how and when genetic tests were used, medical geneticists now called on regulatory agencies, particularly FDA, to ensure that tests were used in ways that benefited rather than harmed those tested. They adopted the language of “clinical utility” to capture this idea of benefit.

Following the history of these regulatory initiatives from the perspective of frame theory thus helps to understand why concerns about the clinical utility of medical tests were first articulated specifically in relation to genetic tests. It also serves to highlight the socio-historical specificity of medical geneticists' concerns. Present-day claims that genetic tests require different regulatory and governance arrangements from other kinds of medical tests—often dubbed “genetic exceptionalism” (e.g. Murray, 1997; Garrison et al., 2019)—are usually justified in terms of the distinctive characteristics of genetic information. In particular, advocates of enhanced regulation of genetic tests argue that they provide information about the present and future health, not only of the individual tested, but also of that individual's genetic relatives; and as such, they pose an especially acute risk of medical and other kinds of discrimination. These were not the concerns that prompted the regulatory initiatives documented in the present paper, however.

From the 1970s onwards, a major part of the practice of medical genetics involved prenatal testing for birth defects including Down syndrome and neural tube defects. In most cases, these conditions do not have a significant familial component. Nonetheless, such tests featured prominently in geneticists' anxieties about genetic testing from the Committee for the Study of Inborn Errors of Metabolism in the 1970s to debates over implementing CLIA88 in the early 1990s. Meanwhile, insofar as medical geneticists also dealt with familial conditions such as cystic fibrosis during this period, they encountered them principally through clinical observation of individual cases and affected families. The testing technologies available at that time served merely to refine what was already known about family history or, in the case of phenylketonuria, to facilitate early diagnosis of what would soon become clinically apparent even in the absence of testing. Consequently, genetic tests revealed little about health or reproductive risks that relatives could not know by other means: family history was informative about genetic tests, rather than vice versa.

Instead, as we have seen, medical geneticists' framing of the dangers of genetic testing focused on the interpretative and inter-personal challenges of communicating complex and often uncertain information about risk, and on the anxiety and suspicion that unnecessary or poorly conducted tests could cause. This remained their principal worry into the early 1990s, evident for instance in Caskey and Lubs's characterisation of genetic tests as having “unique features which require interpretation of data on a highly individualized basis for a specific family or patient” (House Subcommittee on Human Resources and Intergovernmental Relations, 1993, p. 107). As the identification of ΔF508 then other pathogenic mutations made it possible to test for more and more genetic susceptibilities in the absence of any prior knowledge of family history, so concern about the implications of test results for genetic relatives was included within the wider framing of geneticists' anxieties about communicating genetic risk. But their view that genetic tests pose exceptional informational risks far predates the kinds of technical capabilities, and specifically the ability to reveal previously unknown information about family members, that are currently held to justify genetic exceptionalism. Instead, it can be traced back to the early 1970s and the particular predicament in which medical geneticists, eager to expand their practice while distancing themselves from eugenics, found themselves at that time.

Historical framing analysis thus alerts us to how the concerns of the past may inform the present in ways not immediately visible to contemporary actors. At the same time, it also illuminates the limits of that shaping, particularly as ideas and actions are adopted beyond their frame of origin. This is evident from the way the idea of clinical utility has actually been implemented in relation to genetic tests in the years since 2000. Official adoption of medical geneticists' calls for genetic testing to be made conditional on evidence of clinical utility has been piecemeal at best, despite a huge increase in both the range of tests available and the complexity of the genetic information they produce. The Office of Public Health Genomics, established in 1997 as a specialist office of the CDC, incorporated the recommendation into its ACCE (Analytic validity, Clinical validity, Clinical utility and Ethical, social and legal implications) framework in 2000 (Centers for Disease Control, n.d.; Haddow and Palomaki, 2003). Subsequently rolled out under the Evaluation of Genomic Applications in Practice and Prevention (EGAPP) initiative (Teutsch et al., 2009), ACCE assessment has been important in shaping genetic testing as an instrument of public health surveillance (Green et al., 2015). By contrast, FDA has continued to resist taking responsibility for regulating genetic tests (Javitt, 2007), with one partial exception. In 2006, FDA issued draft guidance on regulating a specific class of laboratory-developed genetic tests: so-called in vitro diagnostic multivariate index assays (IVDMIAs). However, Pascale Bourret and colleagues have argued that FDA's embrace of clinical utility as a criterion for assessing IVDMIAs was principally due to researchers' need to standardise outcome measures for clinical trials of novel “personalised” therapies (Bourret et al., 2011; Cambrosio et al., 2017), rather than a concern to protect patients from the risks of genetic testing. This is consistent with FDA's reinterpretation of its regulatory remit as being to foster innovation—for which IVDMIAs hold particular promise—as much as to control the market for medical products (Hogarth, 2015). But it also points to a wider issue surrounding the idea of “clinical utility”.

Neither the Task Force nor the SACGT specified just what kind of evidence would serve to demonstrate the clinical utility of genetic tests. Even the expectation that it include evidence of benefit to those tested, implicit in the committees' overall framing of the risks attending genetic testing, was rarely stated overtly. As the idea of assessing genetic tests for clinical utility was adopted beyond the confines of the policy committees, so it escaped that distinctive framing and began to acquire different connotations. Most notably, the emphasis on assessing harms and benefits to the individuals undergoing testing was progressively weakened. Thus CDC's EGAPP initiative called for “evidence that test results can change patient management decisions and improve net health outcomes (clinical utility)” (Teutsch et al., 2009, p. 6). In the case of FDA's regulation of IVDMIAs, it appears that utility refers even more narrowly to the ability to inform doctors' treatment decisions, especially in clinical research settings (Bourret et al., 2011; Cambrosio et al., 2017).

Meanwhile other organisations, including professional bodies and healthcare providers, have enacted more pervasive regulation of genetic tests through informal governance arrangements such as clinical practice guidelines and health technology assessment procedures (Pitini et al., 2018). While many of these organisations have incorporated clinical utility into their assessment criteria, they varied in just how they evaluated utility (Bossuyt, 2011), with clinical and economic effectiveness often taking precedence over evidence of benefit to individual patients or improved patient outcomes (Smart, 2006). Indeed, of the many genetic tests that have since made their way into clinical practice, many do not even offer improved health outcomes—a case in point being the “arms race” between companies competing to sell tests for more and more cystic fibrosis gene variants, despite the uncertain clinical significance of many of those variants (Grody et al., 2007). In this regard, the proliferation and increasing complexity of genetic tests and the information they provide is probably best seen, not as exceptional, but if anything as exemplary of a more general movement to place data production and management at the heart of modern healthcare.

Faced with these changing conditions of practice, as well as the growth of direct-to-consumer genetic tests of little or no medical value, some social scientists have begun to argue that the concept of “utility” should be expanded to include the public good arising from research conducted on the genetic data accumulated by such means (Turrini and Prainsack, 2016; Haeusermann et al., 2017). This new idea of utility, with its assumption that genetic tests can serve the public good independently of delivering individual benefits, stands in marked contrast to medical geneticists' earlier framing of the potential harms of genetic tests, and especially their insistence that tests be delivered in ways that prioritised the needs and wishes of those tested. In their efforts to repudiate eugenics, those geneticists insisted in effect that risk of harm to individuals should outweigh any claims regarding public benefit. While present-day commentators envisage very different kinds of public good from eugenics, the question of how to balance such goods against individual harms remains pressing—perhaps even more so as the possible harms of genetic testing appear to slip off the regulatory agenda.

## CRediT authorship contribution statement

**Steve Sturdy:** Conceptualization, Investigation, Methodology, Writing - original draft, Writing - review & editing.

## Acronyms used

ASHG: American Society of Human Genetics
CDC: Centers for Disease Control
CLIA88: Clinical Laboratory Improvement Amendments 1988
CSIEM: Committee for the Study of Inborn Errors of Metabolism
DOE: Department of Energy
FDA: Food and Drug Administration
HCFA: Health Care Financing Administration
IOM: Institute of Medicine
NIH: National Institutes of Health
SACGT: Secretary's Advisory Committee on Genetic Testing
